# Characterization of the Tomato *ARF* Gene Family Uncovers a Multi-Levels Post-Transcriptional Regulation Including Alternative Splicing

**DOI:** 10.1371/journal.pone.0084203

**Published:** 2014-01-10

**Authors:** Mohamed Zouine, Yongyao Fu, Anne-Laure Chateigner-Boutin, Isabelle Mila, Pierre Frasse, Hua Wang, Corinne Audran, Jean-Paul Roustan, Mondher Bouzayen

**Affiliations:** 1 Université de Toulouse, INP-ENSA Toulouse, Génomique et Biotechnologie des Fruits, Castanet-Tolosan, France; 2 INRA, Génomique et Biotechnologie des Fruits, Auzeville, France; NIGMS, NIH, United States of America

## Abstract

**Background:**

The phytohormone auxin is involved in a wide range of developmental processes and auxin signaling is known to modulate the expression of target genes via two types of transcriptional regulators, namely, Aux/IAA and Auxin Response Factors (ARF). ARFs play a major role in transcriptional activation or repression through direct binding to the promoter of auxin-responsive genes. The present study aims at gaining better insight on distinctive structural and functional features among ARF proteins.

**Results:**

Building on the most updated tomato (*Solanum lycopersicon*) reference genome sequence, a comprehensive set of *ARF* genes was identified, extending the total number of family members to 22. Upon correction of structural annotation inconsistencies, renaming the tomato ARF family members provided a consensus nomenclature for all *ARF* genes across plant species. *In silico* search predicted the presence of putative target site for small interfering RNAs within twelve *Sl-ARFs* while sequence analysis of the 5′-leader sequences revealed the presence of potential small uORF regulatory elements. Functional characterization carried out by transactivation assay partitioned tomato ARFs into repressors and activators of auxin-dependent gene transcription. Expression studies identified tomato *ARFs* potentially involved in the fruit set process. Genome-wide expression profiling using RNA-seq revealed that at least one third of the gene family members display alternative splicing mode of regulation during the flower to fruit transition. Moreover, the regulation of several tomato ARF genes by both ethylene and auxin, suggests their potential contribution to the convergence mechanism between the signaling pathways of these two hormones.

**Conclusion:**

All together, the data bring new insight on the complexity of the expression control of *Sl-ARF* genes at the transcriptional and post-transcriptional levels supporting the hypothesis that these transcriptional mediators might represent one of the main components that enable auxin to regulate a wide range of physiological processes in a highly specific and coordinated manner.

## Introduction

The plant hormone auxin, indole-3-acetic acid (IAA), is a simple signaling molecule that plays a critical role in plant development and growth. This phytohormone regulates cell division and cell elongation and exerts pleiotropic effects on a wide range of developmental processes including organ differentiation, embryogenesis, lateral root initiation, apical dominance, gravitropism and phototropism, leaf elongation, shoot architecture and fruit development [1], [2], [3], [4], [5]. A critical move towards understanding the mechanisms underlying auxin action [6] happened when it was shown that the hormone coordinates plant development essentially through transcriptional regulation of genes such as *Aux/IAA*, *Gretchen Hagen3* (*GH3*), *Small Auxin Up RNA* (*SAUR*) and *Auxin Response Factor* (*ARF*). It was subsequently found that these so-called early auxin-responsive genes contain in their promoter one or more copies of a conserved motif, TGTCTC or its variants, known as the auxin-responsive element (AuxRE) [7]. Experimental evidences were then provided showing that transcription factors from the ARF type specifically bind to this AuxRE to mediate the transcription of auxin responsive genes [8]. The components of the pathway linking auxin perception to gene expression are now well established indicating that ubiquitination of Aux/IAA proteins by the TIR1/AFB subunit of the SCF^TIR1/AFB^ ubiquitin ligase leads to their degradation by the 26S proteasome thus releasing the Aux/IAA-mediated inhibition of ARFs which allows these transcription factors to modulate the expression of their target genes [9].

Three types of transcriptional regulators are required for the control of auxin-responsive genes, Auxin Response Factors (ARFs), Aux/IAAs and Topless (TPLs) [10], [11]. Members of the Aux/IAA and TPL families have been reported to function as repressors of auxin-induced gene expression [10], [12], [13], [14]. An increasing number of studies demonstrate the critical role of ARFs in a variety of developmental processes, such as embryo patterning [15], [16], leaf expansion and senescence [17], [18], [19], lateral root growth [18], [20], [21], floral organ abscission and petal growth [19], [22], fruit set and development [23], [24], [25], [26], apical hook formation [27], and various responses to environmental stimuli [28]. In addition, *ARF* genes are involved in the cross-talk between auxin and other hormones like gibberellins [29], ethylene [30], ABA [31] and brassinosteroid signaling [32]. A typical ARF protein consists of a conserved N-terminal B3-type DNA Binding Domain (DBD) that regulates the expression of early auxin response genes, a variable middle region (MR) that function as a transcriptional activation or repression domain (AD or RD), and a conserved C-terminal dimerization domain (CTD) that contributes to the formation of either ARF/ARF homo- and hetero-dimers or ARF/Aux-IAA hetero-dimers [8], [33], [34]. The amino acid composition of MRs, located between the DBD and CTD, showed that AD types are rich in glutamine(Q), serine (S), and leucine (L) residues while RD types are rich in proline (P), serine (S), threonine (T), and glycine (G) residues [33], [35].

Since the cloning of the first *AtARF1* from Arabidopsis, 22 members of this family, distributed over all five chromosomes, have been identified [33]. The functional characterization of *AtARF* genes was revealed by mutant analysis approach. For instance, *arf1* and *arf2* T-DNA insertion mutations indicated that ARF2 regulates leaf senescence [17] and floral organ abscission [19]. The *arf7/arf19* double mutant had stronger auxin resistance than the single mutant and displayed phenotypes not seen in the single mutant [30]. ARF8 was reported to regulate fertilization and fruit development, and *arf8-4* mutation results in the uncoupling of fruit development from pollination and fertilization giving rise to parthenocarpic fruit [23], while flowers in *arf6/arf8* double mutant are arrested as infertile closed buds with short petals, short stamen filaments, undehiscent anthers and immature gynoecia [36]. In tomato, recent studies have shown the involvement of *ARF* genes in fruit set, development, ripening and fruit quality [3], [4], [5], [24], [25], [26], [37]. Because of these findings, members of this gene family are becoming one of the main targets towards improving fruit traits in tomato and more broadly in fleshy fruits.

Studies using different species have indicated a total of 25 ARF genes in rice (*Oryza sativa*), 39 ARF genes in *Populus trichocarpa*, 24 ARF genes in sorghum (*Sorghum vulgare*) and 31 ARF genes in maize [38], [39], [40], [41]. Though 21 *ARF* genes have been previously identified in the tomato (*Solanum lycopersicum*), yet, the list was incomplete and some the family members were either misannotated or suffered structural inconsistency due to the lack at that time of a high quality assembled tomato genome sequence [42], [43]. The present study, while comprehensively revising the entire *ARF* gene family in tomato, brings new insight on the complexity of their expression control at the post-transcriptional level. The distinctive spatio-temporal pattern of expression of tomato *ARF* genes and their differential responsiveness to auxin and ethylene lay the foundation for a deeper functional characterization of these transcriptional mediators.

## Results

### Genome-wide search for tomato ARF genes

**Figure 1:**
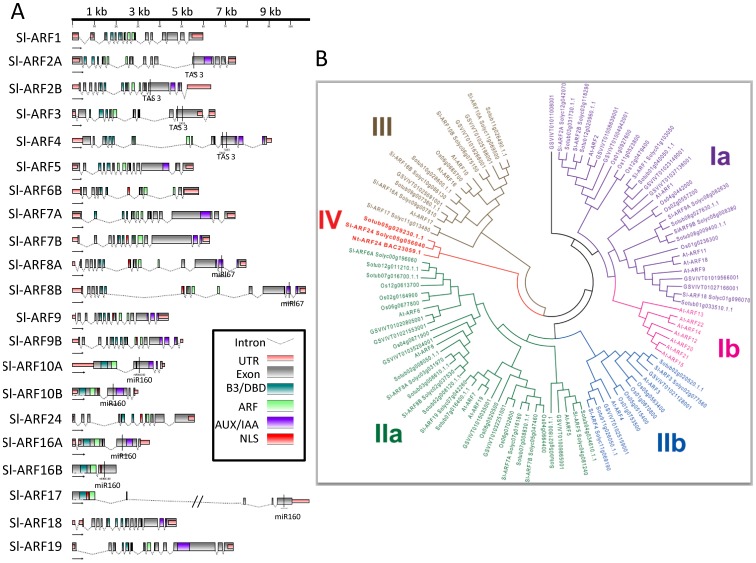
The ARFfamily structures in tomato and phylogenetic relationship between rice, potato, tomato, grape and Arabidopsis. (A) The generic structures of Sl-ARF family except *Sl-ARF6A*. The gene size (kb) is indicated in the upper panel. The domain of Sl-ARF gene is indicated by different colours. The marker in Sl-ARF family shows*Sl-ARF2A, 2B, 3* and *4*genes are spliced by TAS 3, *Sl-ARF8A* and *8B* spliced by miRl67, and *Sl-ARF10A, 10B, 16A, 16B* and *17* spliced by miR160.(B) The unrooted tree was generated using MEGA4 program by neighbor-joining method. Bootstrap values (above 50%) from 1000 replicates are indicated at each branch. All Sl-ARFs contain a DBD (brown). Most of the Sl-ARF proteins except Sl-ARF3, 10, 24, 16 and 17 contain a carboxy-terminal domain related to the domains III and IV found in the Aux/IAA proteins (blue).Sl-ARF5, 6A, 7, 8A, 8B, 19 contains a middle region that corresponds to the predicted activation domain (green) found in some AtARFs. The remaining Sl-ARFs contains a predicted repression domain (red). Sl-ARF-6B and AtARF23 contain only a truncated DBD (B3 domain).

Comprehensive identification of the *ARF* gene family members in the tomato was achieved using all ARF proteins previously reported from Arabidopsis and other plant species in BLAST queries on the recently published tomato genome sequence (SL2.40 genome sequence and iTAG2.30 whole protein sequences). Twenty four significant hits corresponding to non-redundant putative *Sl-ARF* genes were identified. PCR amplification of full length coding sequences (CDS) revealed two structural annotation inconsistencies reducing the total number of ARFs in the tomato genome to 22 (Table 1). Indeed, four sequences previously annotated as distinct *ARF* genes in iTAG2.30 corresponded to C-terminal or N-terminal parts of two ARF proteins (Solyc12g006340/Solyc00g196060; Solyc11g013480/Solyc11g013470). The mapping of tomato RNA-Seq data allowed to further improve the annotation of tomato *ARFs* by identifying the 3′ and/or 5′ UTR regions for 13 *Sl-ARF* genes (Table 1). All Sl-ARF proteins were found to contain a typical DBD domain (Figure 1A) as revealed by the Pfam analysis tool (http://pfam.sanger.ac.uk/). The molecular weight of the deduced Sl-ARF proteins showed a large variation ranging from 68 to 126 kDa (Table 1). Of particular note, *Sl-ARF6B* contains a premature stop codon in the region corresponding to the DBD domain and is therefore likely to be a pseudo-gene whose expression at the protein level is not expected (data not shown). Using cNLS Mapper, nuclear localisation signals (NLS) were also identified in all of Sl-ARFs (data not shown).

**Table 1:** *Sl-ARF* gene family in tomato.

Generic name^a^	Alias^b^	CDS length^c^	Str	Chr	Domains^d^	Name in Wu et al.^e^	improvement^f^	New location^g^
Sl-ARF1	Solyc01g103050	1965	+	1	B3,ARF,AUX/IAA,SPL-Rich RD	SlARF1(HM061154.1)	-	
Sl-ARF2A	Solyc03g118290	2541	+	3	B3,ARF,AUX/IAA,SPL-Rich RD	SlARF2(DQ340255.1)	-	
Sl-ARF2B	Solyc12g042070	2490	-	12	B3,ARF,AUX/IAA,SPL-Rich RD	SlARF11(HM143940.1)	5′, 3′ UTR	42538600..42544937
Sl-ARF3	Solyc02g077560	2244	+	2	B3,ARF,SL/G-Rich RD	SlARF3(DQ340254.1)	-	
Sl-ARF4	Solyc11g069190	2436	-	11	B3,ARF,AUX/IAA,SPL-Rich RD	SlARF4(DQ340259.1)	5′, 3′ UTR	50900912..50910023
Sl-ARF5	Solyc04g081240	2793	-	4	B3,ARF,AUX/IAA,QSL-Rich AD	SlARF5(HM195248.1)	-	
Sl-ARF6A	Solyc12g006340(Nter);Solyc00g196060(Cter)	2643	−/+	12/0	B3,ARF,AUX/IAA,QSL-Rich AD	SlARF6(HM594684.1)	5′ UTR, CDS	857256..859656(Nter)
Sl-ARF6B	Solyc07g043620	2673	-	7	B3,ARF,QSL-Rich AD	SlARF6-1(NM_001247611.1)	5′UTR	54884781..54890560
Sl-ARF7A	Solyc07g016180	3339	-	7	B3,ARF,AUX/IAA,QSL-Rich AD	SlARF19(HM130544.1)	-	
Sl-ARF7B	Solyc05g047460	3294	-	5	B3,ARF,AUX/IAA,QSL-Rich AD	SlARF19-1(HM565130.1)	5′UTR	58050744..58057040
Sl-ARF8A	Solyc03g031970	2535	+	3	B3,ARF,AUX/IAA,QSL-Rich AD	SlARF8-1(HM560979.1)	5′UTR	8739535..8747501
Sl-ARF8B	Solyc02g037530	2529	+	2	B3,ARF,AUX/IAA,QSL-Rich AD	SlARF8(EF66734F2.1)	5′UTR	21756022..21766699
Sl-ARF9A	Solyc08g082630	1977	+	8	B3,ARF,AUX/IAA,SPL-Rich RD	SlARF9(HM037250.1)	5′UTR	62527409..62531812
Sl-ARF9B	Solyc08g008380	2052	+	8	B3,ARF,AUX/IAA,SPL-Rich RD	SlARF12(HM565127.1)	5′UTR	2807931..2812983
Sl-ARF10A	Solyc11g069500	2100	-	11	B3,ARF,AUX/IAA,SL/G-Rich RD	SlARF10(HM143941.1)	5′, 3′ UTR	51188434..51192539
Sl-ARF10B	Solyc06g075150	2016	+	6	B3,ARF,AUX/IAA,SL/G-Rich RD	SlARF16(HM195247.1)	3′ UTR	43020594..43023604
Sl-ARF24	Solyc05g056040	1953	-	5	B3,ARF,SPL-Rich RD	SlARF13(HM565128.1); SlARF13-1(HM565129.1)	-	
Sl-ARF16A	Solyc09g007810	2085	-	9	B3,ARF,AUX/IAA,SL/G-Rich RD	No	3′ UTR	1332230..1335760
Sl-ARF16B	Solyc10g086130	1896	-	10	B3,ARF,SL/G-Rich RD	SlARF16(NM_001247861.1)	-	
Sl-ARF17	Solyc11g013480(Nter);Solyc11g013470(Cter)	1869	-	11	B3,ARF,SL/G-Rich RD	SlARF17(HQ456923)	3′ UTR, CDS	6495469..6511349
Sl-ARF18	Solyc01g096070	2058	+	1	ARF,AUX/IAA,SPL-Rich RD	No	5′UTR	78941268..78946012
Sl-ARF19	Solyc07g042260	3357	-	7	B3,ARF,AUX/IAA,QSL-Rich AD	SlARF7(EF121545.1)	-	

^a^ Sl-ARF gene names

^b^ the alias of each *ARF* gene in iTAG2.30 genome annotation

^c^ Length of the corresponding Coding Sequence (CDS) in base pairs.

^d^ Conserved Domains found in PFAM database: B3 means DNA binding domain, ARF means Auxin response Factor conserved domain, AUX/IAA means AUX/IAA dimerization domain, AD means transcriptional activation domain, RD means transcriptional repression domain.

^e^ Corresponding names in Wu et al.; accession numbers are in the parentheses.

^f^ Gene Model modification type: UTR means that the UTR sequence have been identified and annotated, CDS means that the the coding sequence have been corrected.

^g^ New locations in the tomato genome version Sl2.40 taking into account the manual curation of the previous gene annotation in iTAG2.30

Building on the available tomato genome assembly sequence, the mapping of *Sl-ARF* genes revealed that *Sl-ARF* family members are distributed among the 12 tomato chromosomes. Chromosome 7 and 11 are found to harbor three *ARF*genes each; chromosome 1, 2, 3, 5, 8 and 12 bear two *ARFs*, while each of chromosome 4, 6, 9 and 10 contains only a single *ARF* gene (Figure S1 in File S1). Unlike the situation prevailing in Arabidopsis, there is no evidence for tandem or segmental duplication events involving members of the tomato ARF family.

### Phylogenetic relationship and consensus nomenclature for Sl-ARFs

To explore phylogenetic relationship among ARF proteins in largely distributed land plant species, a phylogenetic tree (Figure 1B) was constructed that included ARF family members from tomato, *Arabidopsis*, potato, grape and rice. The phylogenetic distribution revealed that *ARF* genes group into four major classes named Class I, II, III and IV. ARFs predicted to function as transcriptional activators, based on the presence the Q-rich activation domain in their middle region, belong to sub-class IIa (Sl-ARF5, Sl-ARF6A, Sl-ARF7A, Sl-ARF7B, Sl-ARF8A, Sl-ARF8B and Sl-ARF19) while ARFs from the remaining classes (Ia, IIb, III and IV) all harbor a repression domain in the middle region and are consequently predicted to function as transcriptional repressors.

Compared to Arabidopsis which contains 23 members, the size of the tomato *Sl-ARF* gene family is slightly contracted to 22 members. In order to reach a consensual nomenclature for *ARF* genes across species, the tomato members of this gene family were renamed, based on phylogenetic relationship and according to the numbering of the closest Arabidopsis homolog. While complying with the most complete classification available in Arabidopsis [33], the proposed nomenclature better clarifies the correspondence between ARF subclasses in plant species. Noteworthy, sub-class Ib which has no representative in the tomato, contains 7 members in Arabidopsis that are likely to derive from multiple duplications of At-ARF13 which has no ortholog in any of the plant species tested in the present study. A distinctive feature of the tomato ARF family is the expanded size of the activators' sub-class (IIa) which represents 36.5% of the *ARF* genes whereas the activators only account for 21.7% of Arabidopsis ARFs. Another specific feature of the tomato ARF family is the presence of *Sl-ARF24* (sub-class IV) that is not found out of the *Solanaceae* family. Interestingly, this presumably *Solanaceae*-specific gene encodes an ARF protein that lacks domain III and IV involved in protein/protein interactions and required for the binding to Aux/IAA proteins. Likewise, Sl-ARF3, Sl-ARF16B and Sl-ARF17 are also deprived of domain III and IV necessary for interaction with Aux/IAAs (Figure 1A and Figure S2 in File S1). It is therefore likely that these Sl-ARFs escape the classical mechanism underlying auxin signaling which implies the sequestration of ARF proteins through interaction with Aux/IAAs.

### Predicted siRNA-mediated degradation and multiple upstream ORFs in the 5′ leader sequences of tomato ARF transcripts

ARF genes have been already reported to undergo post-transcriptional regulation involving small interfering RNAs. *In silico* analysis at the RNA level predicted that 12 out of the 22 tomato Sl-ARFs have a putative target site for small interfering RNAs (Figure 1A). That is, Sl-ARF2A, Sl-ARF2B, Sl-ARF3 and Sl-ARF4 are predicted to be potentially targeted by TAS3; Sl-ARF6A, Sl-ARF8A and Sl-ARF8B by miR167; and Sl-ARF10A, Sl-ARF10B, Sl-ARF16A, Sl-ARF16B and Sl-ARF17 by miR160.

The uORFs are elements found in the 5′-leader sequences of specific mRNAs that modulate the translation of downstream ORFs by ribosomal stalling and inefficient re-initiation or by affecting transcript accumulation through nonsense-mediated mRNA decay pathway. Among the 19 *Sl-ARFs* for which the 5′ leader sequences are available in iTAG2.30 (8 members) or identified in this study (11 members), uORFs were predicted for 17 genes, ranging from 1 to 52 amino acids in size with four genes (Sl-ARF2A, Sl-ARF5, Sl-ARF10A and Sl-ARF16A) having five or more uORFs (Table S1 in File S1).The average number of uORF per Sl-ARF gene is similar in tomato (2.8/leader) and Arabidopsis (3.3/leader), indicating that tomato ARFs are suitable candidates to be regulated through translational uORFs depending mechanism

### Transcriptional activation and repression activities of tomato ARFs

**Figure 2:**
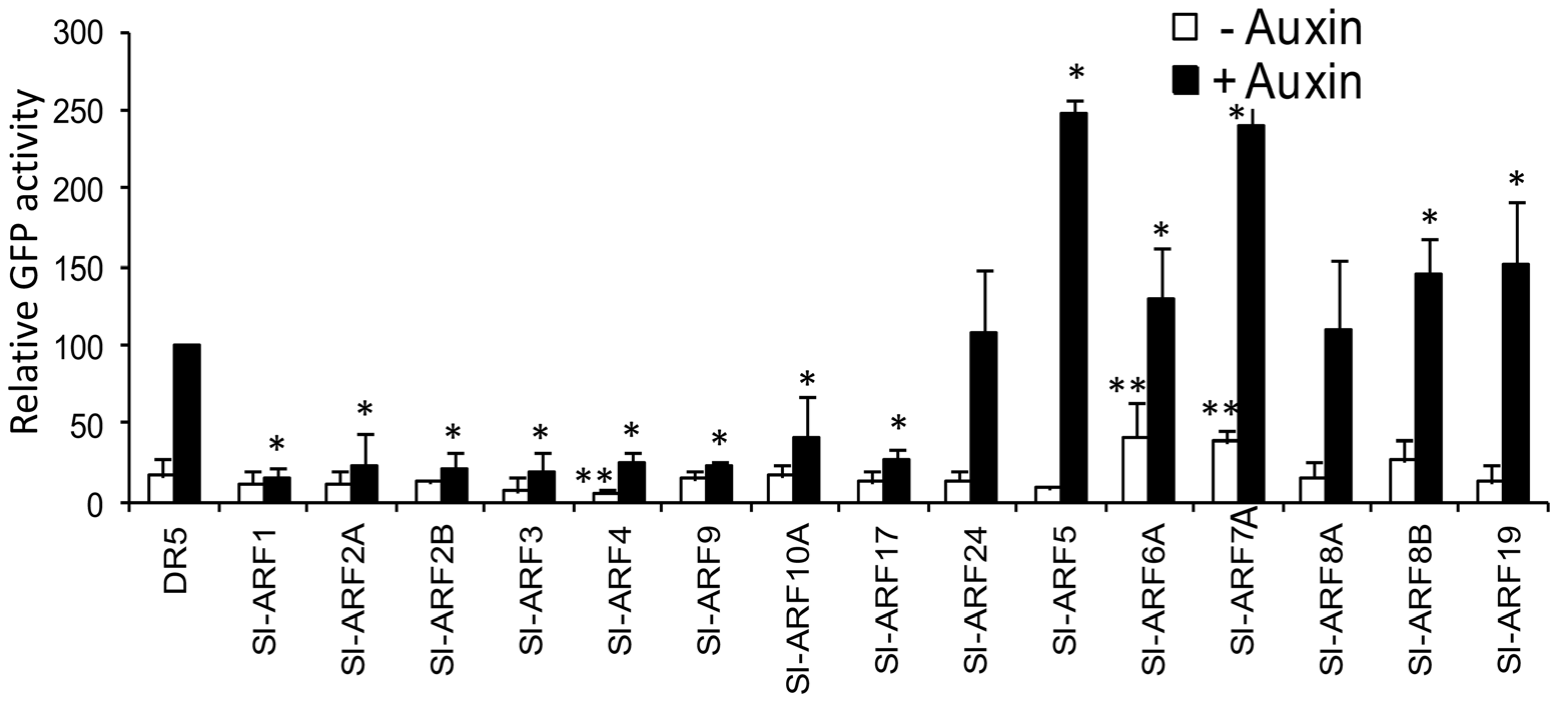
Sl-ARF factors differentially regulate the expression of reporter genes driven by synthetic and native auxin-responsive promoters. Sl-ARF factors were challenged with a synthetic auxin-responsive promoter called *DR5*, consisting of seven tandem copies of the AuxREtgtctc element. A transient expression using a single cell system was performed to measure the reporter gene activity. The fluorescence was measured by flux cytometry. Because of the very low basal activity of the DR5 promoter without auxin treatment, the auxin inducible fluorescence obtained by co-transformation with the promoter fused to the reporter gene and with the empty vector was standardized to 100 and taken as reference. Biological triplicates were averaged and analysed statistically using Student's t-test at (P<0.05). (*) indicates significant changes corresponding to co-transformation with effector Sl-ARF and reporter DR5-GFP constructs compared to basal activity of DR5 promoter in the absence of auxin treatment. (**) indicates significant changes for the same experiment carried out in the presence of auxin Bars indicate the SEM.

To characterize the capacity of tomato ARF proteins to *in vivo* activate or repress gene transcription, tobacco cells were co-transfected with an effector construct expressing the full-length coding sequence of Sl-ARFs and a reporter construct carrying the auxin-responsive *DR5* promoter fused to *GFP* coding sequence [44]. DR5 is a synthetic auxin-responsive promoter made of 9 inverted repeats of the conserved Auxin-Responsive Element, the so-called TGTCTC box, fused to a *CaMV35S* minimal promoter. The *DR5-*driven GFP chimeric gene showed low basal activity which was induced up to 5-fold by exogenous auxin treatment (Figure 2). Co-transfection of tobacco protoplasts with the *DR5::GFP* reporter construct and effector plasmids expressing either *Sl-ARF1, Sl-ARF2A, Sl-ARF2B, Sl-ARF3, Sl-ARF4, Sl-ARF9A, Sl-ARF10A* or*Sl-ARF17* coding sequences, resulted in repression of the auxin-induced expression of the reporter gene (Figure 2). By contrast, co-transfection with effector constructs expressing *Sl-ARF5, Sl-ARF6A, Sl-ARF7, Sl-ARF8B* or *Sl-ARF19* enhanced slightly the auxin-induced expression of the reporter gene. Noteworthy, with the exception of *Sl-ARF6A* and *Sl-ARF7*, these activator ARFs were unable to enhance the basal activity of the DR5 promoter in the absence of auxin treatment (Figure 2) suggesting that most ARFs require the input of an active auxin signalling for transcriptional activation of target genes.

### Expression of Sl-ARF genes in different tomato tissues

**Figure 3:**
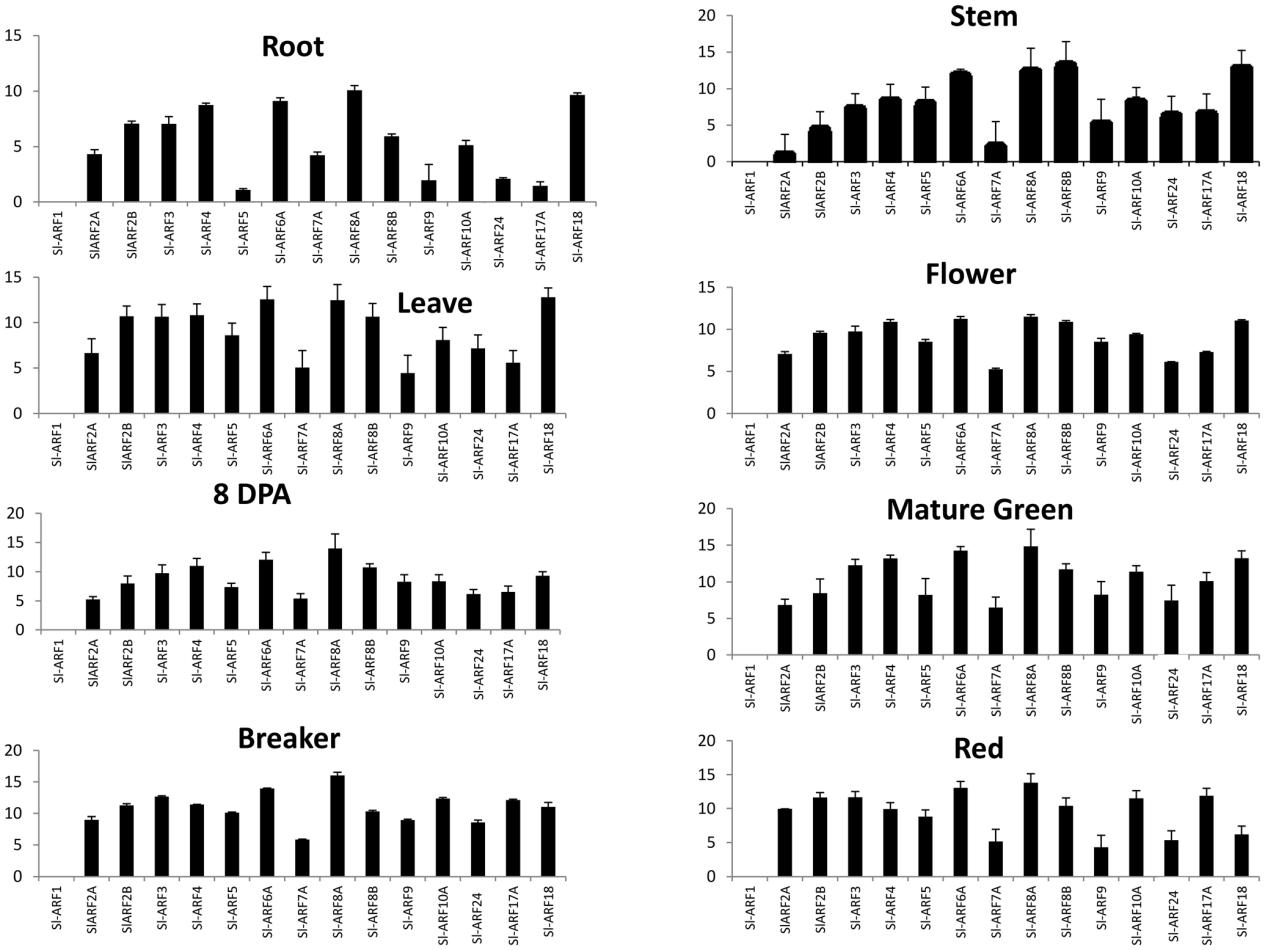
Real-time PCR expression profiles of individual *Sl-ARF* genes. Total of 15 *Sl-ARF*genes were performed in different tomato organs (root, stem, leaf, flower, 8DPA, Mature Green, Breaker and Red). X-axis represents different *Sl-ARF* genes, while Y-axis represents three relative expressions of those genes. 8DPA: 8 days after pollination, Mature Green, Breaker and Red represent different stage of the fruit development.

**Figure 4:**
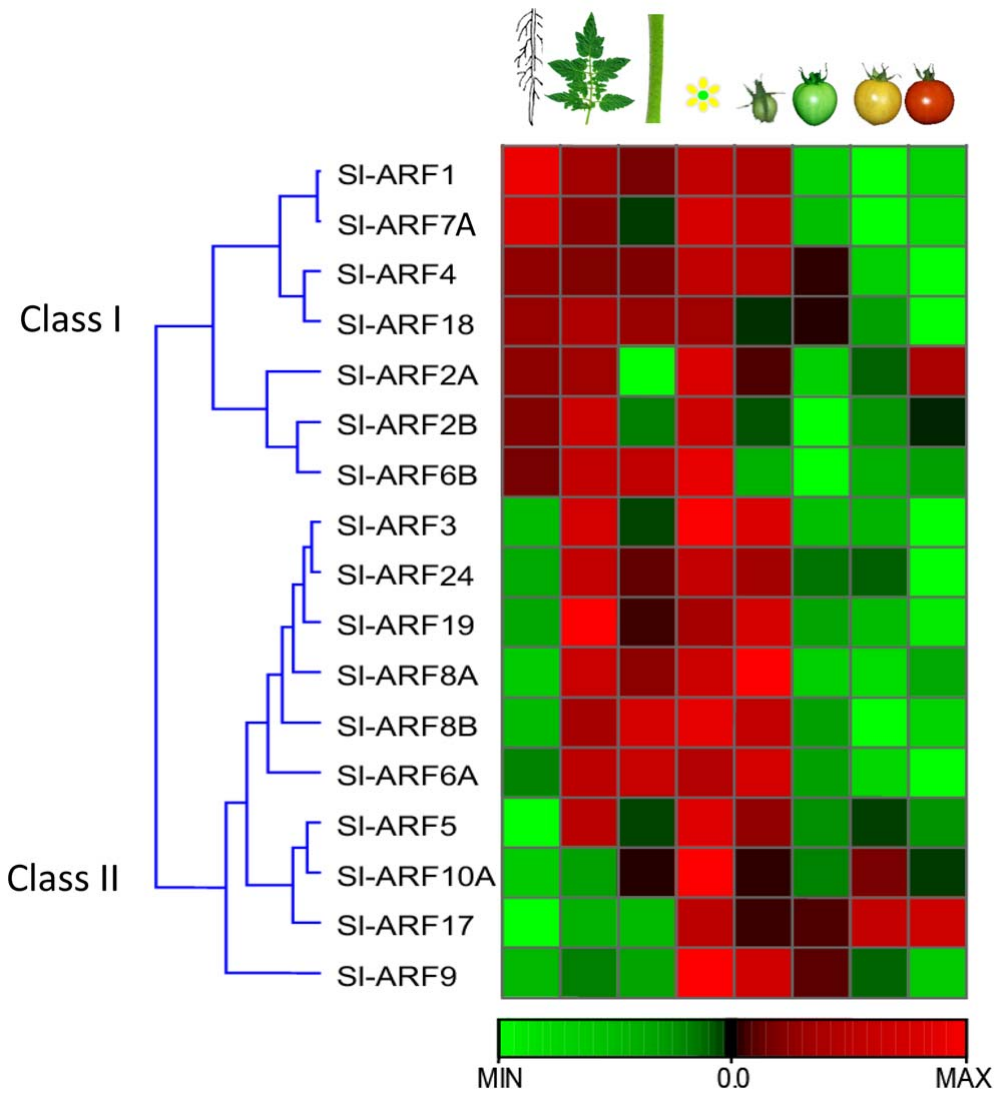
Heatmap showing *Sl-ARF* gene expression in different tomato tissues. Changes in RNA accumulation in different tomato tissues (Roots, Leaves, Stems, Flowers, Early Immature Green (8 DPA), Mature Green, Breaker, Red (Breaker + 7 days) as schematically depicted above the displayed array data, are shown relative to the RNA accumulation levels in roots. Levels of down expression (green) or up expression (red) are shown on a log2 scale from the high to the low expression of each *Sl-ARF* gene.

To gain clues on the physiological function of tomato ARFs, the spatio-temporal expression of individual members of the gene family was examined at the transcriptional level using qRT-PCR. Transcript accumulation could be assessed for 15 *ARF* genes in different tissues including root, stem, leaves, flower and fruit at various developmental stages. For the remaining 7 tomato *ARF* genes, transcript detection was unsuccessful in any of the samples tested suggesting their extremely low expression in these tissues. The data indicate that the expression of *ARF* genes is ubiquitous in all tissues with most genes being expressed in reproductive tissues suggesting their putative role in flower and fruit development (Figure 3). Heatmap representation (Figure 4) allowed the clustering of tomato *ARFs* into two main groups based on their expression pattern: group I (*Sl-ARF1, Sl-ARF2A, Sl-ARF2B, Sl-ARF4, Sl-ARF7A, Sl-ARF6B and Sl-ARF18*) are genes preferentially expressed in roots and group II *Sl-ARFs* in the areal part of the plant.

Sl-ARF6B displays a very low expression in all tomato tissues analyzed and the corresponding CT values showed high variability among repeats making these Sl-ARF6B expression data not meaningful. Therefore, they were not included in Figure 3 but retained for the heat map (Figure 4) in despite of the variability between the repeats in order to give a general idea about its expression in different tissues.

### Auxin and ethylene regulation of Sl-ARF genes

**Figure 5:**
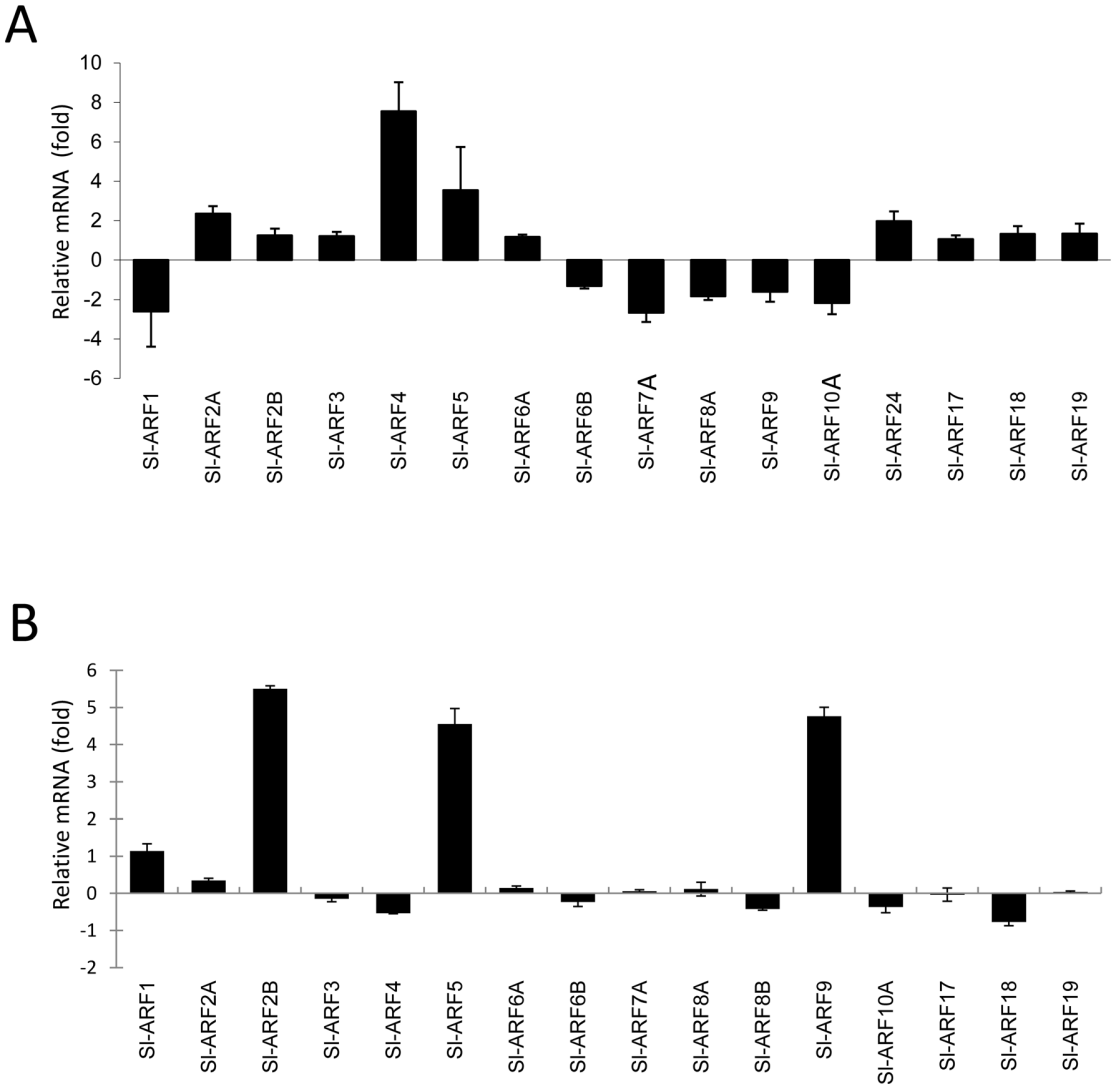
The expression of *Sl-ARF* family genes in response to auxin and ethylene. (A) Auxin induction of *Sl-ARF* genes on light grown seedlings. Quantitative RT-PCR of *Sl-ARF* transcripts in RNA samples extracted from 12-day-old tomato seedlings soaked in liquid MS medium with 10 µM IAA for 2 hours. ΔΔCT refers to the fold of difference in *Sl-ARF* expression to the untreated seedlings. The *SAUR* gene was used as control to validate the auxin treatment.(B) Ethylene regulation of *Sl-ARF* genes on dark grown seedlings. Quantitative RT-PCR of *Sl-ARF* transcripts in RNA samples extracted from5-days dark-grown tomato seedlings treated 5 hours with ethylene (50 µL/L). ΔΔCT refers to fold differences in *Sl-ARF* expression relative to untreated seedlings. The *E4* gene was used as control for efficient ethylene treatment.

Screening for *cis*-acting elements corresponding to Auxin Response Elements (AuxRE) within the promoter regions using the Place database (http://www.dna.affrc.go.jp/PLACE/signalscan.html) identified conserved (TGTCTC) and degenerate (TGTCCC) motifs in most tomato ARF promoters. In addition to these AuxRE, *Sl-ARF* promoters contain conserved Ethylene-Response motifs, the so-called ERELEE4 motif found in the promoter of tomato *E4* gene (AWTTCAAA) (Table S2 in File S1). The presence of these *cis*-regulatory elements suggests a potential regulation of *ARF* genes by both auxin and ethylene. To test the responsiveness of tomato *ARF* genes to both hormones, transcript accumulation was assessed by qRT-PCR in seedlings treated with auxin or ethylene. All *Sl-ARFs* were found to be auxin-responsive after 2 hour treatment (Figure 5A), with *Sl-ARF4*, *Sl-ARF5* and *Sl-ARF2A* showing the highest up-regulation whereas *Sl-ARF1*, *Sl-ARF7 Sl-ARF10* displayed the most significant down-regulation. On the other hand, the expression of Sl-ARF2B, Sl-ARF5 and Sl-ARF9A showed strong up-regulation (more than four folds increase) when treated 5 hours with ethylene (Figure 5B). Of particular interest, Sl-ARF5 is strongly up-regulated by both hormones and may therefore be involved in mediating responses to both hormones.

### Expression of Sl-ARF genes during tomato fruit set

**Figure 6:**
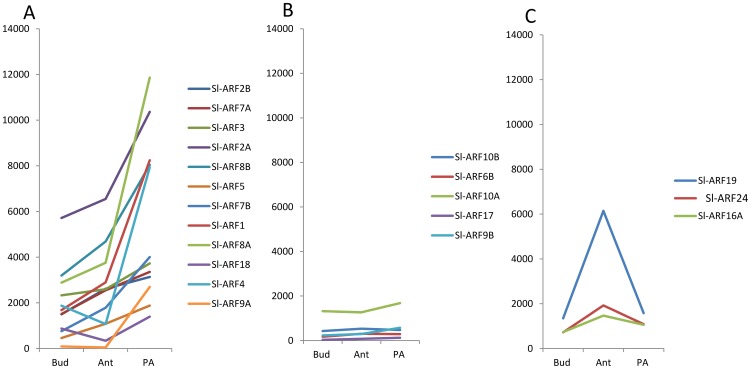
The expression profile of *Sl-ARF* family genes in tomato fruit set. (A)12 *Sl-ARF* genes are over-expressed after pollination and fertilization (4DPA), which are Sl-ARF9A, 4, 18, 8A, 1, 7B, 5, 8B, 2A, 3, 7A and 2B genes in turn according to the log change of P/A (Post-anthesis/Anthesis). (B) 5 *Sl-ARF* genes keep stable expression from flower bud to post-anthesis, includingSl-ARF10A, 10B, 6B, 9B, 17 genes.(C) 3 *Sl-ARF* genes are up-regulated from flower bud to anthesis and down-regulated after pollination and fertilization (4DPA), including Sl-ARF24, 19, and 16A genes. The expression values are taken from RNA-sequencing data and the colors represent different *Sl-ARF* genes.

The expression of a high number of *Sl-ARFs* in reproductive tissues (Figure 3 and 4) along with the previously reported role of auxin in controlling the fruit set process, prompted us to investigate the expression of *Sl-ARF* genes during the flower-to-fruit transition. To determine the expression dynamics throughout the fruit set process, transcript accumulation of tomato *ARFs* was monitored by RNA-seq approach at flower buds, anthesis and pos-anthesis stages (young fruit at 4 DPA). For each stage, RNA libraries were generated from three independent biological replicates and subjected to Illumina mRNA-Seq technology sequencing (Data desposited at NCBI SRA database under accession number SRP029978). Reads were then mapped on the tomato genome sequence and read counts were determined as described in Maza et al. 2013 [45]. The data indicate that most *Sl-ARFs* undergo a strong change in their expression associated with the flower-to-fruit transition (Figure 6). Three groups could be discriminated based on RNA counts distribution during the fruit set process. Group 1 corresponds to *Sl-ARFs* whose expression increased following pollination, Group 2 to *ARFs* with unchanged expression and Group 3 to *Sl-ARFs* displaying decreased expression following pollination (Figure 6).

### Sl-ARF transcripts undergo intense alternative splicing during tomato fruit set

**Figure 7:**
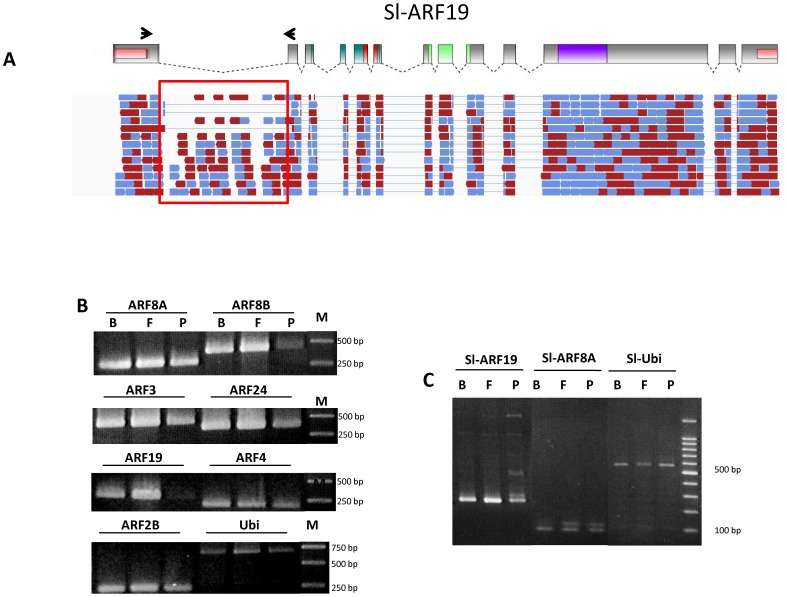
The ARF family genes showed alternative spilcing mode of regulation in tomato fruit set. (A) RNA-seq reads generated during the fruit-set and mapped on *Sl-ARF19* gene structure showing one alternative spicing that can be generated in the Intron 1. Reads are represented by red and blue rod arrows (B) The RT-PCR was carried out using pairs of primers designed within the introns of 7 *Sl-ARF* genes highlighted in Figures S3.1 to S3.6 in File S1, such as Sl-ARF8A_Intron 6, Sl-ARF8B_Intron 11, Sl-ARF3_Intron 9, Sl-ARF24_Intron 3, Sl-ARF19_Intron 1, Sl-ARF4_Intron 6 and Sl-ARF2B_Intron 11. The ubiquitin gene was used as the reference. (C) The RT-PCR was performed using pairs of primers nested in the two exons encompassing the intron of target Sl-ARF genes, such as Exon1-Exon2 in Sl-ARF19 and Exon6-Exon7 in Sl-ARF8A. The cDNAs generated from flower bud (B), flower at anthesis (F) and young fruit 4 days post-pollination (P) tissues were used as the template. The ubiquitin was used as the reference.

Closer analysis of the mapping of RNA-seq data on the gene models revealed possible alternative splicing regulation during fruit set for 30% of *Sl-ARF* genes. *Sl-ARF2B* and *ARF19* shows one possible alternative splicing occurring at intron 11 and intron 1, respectively (Figure 6A and Figures S3.1 in File S1). *Sl-ARF3* and *Sl-ARF4* could putatively give rise to two alternative splicing events at introns 7 and 9, and at introns 6 and 10, respectively. Three possible alternative splicings were found at introns 3, 6 and 10 in *Sl-ARF8A* and at introns 9, 11 and 13 in *Sl-ARF8B*. Finally, *Sl-ARF24* offers up to four alternative splicing possibilities at introns 1, 3, 6 and 10 (Figures S3.1–6 in File S1). In all cases, the detected Sl-ARF splice variants resulted in a frame shift within the coding region that generates a premature stop codon. To further validate the occurrence of the alternative splicing forms and assess the relative levels of the various splice variants, a semi quantitative PCR approach was conducted. To this purpose, two pairs of primers were designed, one aiming to specifically amplify the retained intron fragment while the second pair was designed in the margins of the two exons framing the retained intron. A PCR product with the expect size was detected for all genes confirming the presence of the splice variant in each RNA extraction (Figure 7B). Interestingly, the data indicate that the abundance of the *Sl-ARF8B_int11* transcript variant decreases dramatically in young fruits whereas the global expression of the corresponding *Sl-ARF8B* gene increases significantly. This finding suggests that the down-regulation of the *Sl-ARF8B_int11* transcript variant may potentially play a role in the regulation of the flower to fruit transition. By contrast, increased accumulation of the *Sl-ARF19_int1* was observed concomitant to the transition from flower to fruit. Taking together, these data uncover a potential role for alternative splicing in regulating the expression of tomato *ARFs* during the fruit set process.

## Discussion

Being down-stream components of auxin signalling pathway, ARFs likely contribute to the specificity of the hormone responses. Hence, the functional characterization of these transcriptional mediators is essential towards understanding the mechanisms by which auxin triggers appropriate growth and developmental responses in a timely and tissue-specific manner. To better define the role of ARFs in mediating specific auxin responses, the present study brings a complete picture on the main structural features of the tomato *ARF* gene family. Identification of tomato *ARFs* has been already described but this attempt built on a draft tomato genome sequence and ESTs and could therefore not be comprehensive [42], [43]. The present work takes advantage of the most updated tomato reference genome sequence [46] to isolate the complete ARF family members and perform functional analysis and expression profiling of these transcriptional regulators. Using these extended resources, the list of tomato *ARFs* has been enlarged to 22 members and manual annotation based on deep RNA-Seq data, allowed the curation of some structural annotation inconsistencies as well as the identification of the 3′ and 5′ UTR regions for more than 50% of the *Sl-ARF* gene family. The tomato members of the ARF family were renamed according to the numbering of the closest Arabidopsis homolog, which provides a consensus nomenclature for *ARF* genes across plant species. In this way, the proposed nomenclature better clarifies the correspondence between ARF subclasses in various plant species. The phyllogenetic approach applied on a well distributed set of plant ARFs allowed to identify a specific sub-class (sub-class IV) that is absent out of the *Solanaceae* family. Interestingly, this sub-class contains a specific gene, *Sl-ARF24*, encoding a putative ARF protein that lacks the two protein/protein interaction domains, known as domain III and IV and required for the binding to Aux/IAA proteins. It is therefore likely that Sl-ARF24 escapes the classical mechanism underlying auxin signaling which implies the sequestration of ARF proteins through interaction with Aux/IAAs.

As a preliminary approach towards functional characterization of members of the tomato ARF family, the present study describes their expression pattern, their post-transcriptional regulation and their ability to activate or repress transcriptional activity on synthetic or native auxin-responsive promoters. Transactivation assays revealed that 36% of tomato ARFs are strong repressors of transcriptional activity while only 22% are transcriptional activators. The repressor/activator ratio among ARFs is more than twice higher in tomato (3.6) compared to Arabidopsis (1.7), yet, it remains to be elucidated whether this feature may account for differences in developmental and growth behaviour between the two species. In contrast to repressor ARFs, most activator Sl-ARFs promote transcription of target genes only upon exogenous auxin treatment thus suggesting that activator ARFs require some input from a highly activated auxin signalling pathway in order to potentiate transcriptional activity. It is conceivable that when the auxin level is low, the amount of Aux/IAA proteins available is sufficient to block ARFs at the protein level thus preventing these latter from activating the transcription of the target genes. In this perspective, it has to be postulated that Aux/IAAs are present in excess in the cell when the tissue is not subjected to auxin treatment.

The spatio-temporal pattern of expression indicated that all *Sl-ARF* genes are expressed in flower and fruit suggesting a putative important role in reproductive tissue development. The shift from the static flower ovary to fast-growing young fruit is a phenomenon known as fruit set and auxin has been shown to play a crucial role in controlling this developmental process [47], [48] representing an important step in the development of all sexually reproducing higher plants. Adding to the primary role of Aux/IAAs in triggering the fruit set process previously reported [3], [49], the present study reveals the potential active role of a number of Sl-ARFs during this process based on genome-wide transcriptomic profiling of the flower to fruit transition. The expression of 12 members of the gene family sharply increases upon pollination/fertilization, while the expression of a fewer number of Sl-ARF genes peaks at anthesis and then dramatically declines at post-pollination stage. Given the role of auxin signaling in the fruit set process [48], [50], the dynamics of the expression pattern of these Sl-ARFs is indicative of their putative involvement in mediating auxin responses during the flower-to-fruit transition. This is consistent with the prominent role reported for *Sl-ARF8A* and *Sl-ARF7* (referred as Sl-ARF19 in the present study) during fruit set and parthenocarpy in Arabidopsis and tomato, respectively [24], [26]. Of particular interest, *Sl-ARF8A* shows the most dramatic rise in expression at post-anthesis stage which may designate this ARF among all family members as the main actor of the fruit set process.

The data indicate that tomato ARFs are subject to multi-levels post-transcriptional regulation of their expression. In line with Arabidopsis ARFs [51], [52], [53], it is shown here that 11 out of the 22 tomato *ARF* genes are potentially regulated by siRNAs. Moreover, the direct evidence for active alternative splicing described here uncover a new layer of complexity in the post-transcriptional regulation of ARF genes in the tomato. This mode of regulation may account for a significant part of the control of ARF expression in developmental processes such as fruit set in the tomato as indicated by the abundance of some transcript splice variants concomitant to the flower to fruit transition. An additional mean towards controlling ARF expression in the tomato may also take place at the translational level via upstream ORFs (uORFs) that have been predicted in most members of *ARF* genes. This mode of regulation has been first suggested in Arabidopsis where *in silico* search revealed an enrichment of uORFs in the ARF 5′-leader sequences that is not seen in other auxin-related genes such *Aux/IAA*, *YUCCA*, *TIR1* auxin receptors homologs and *PIN* family of auxin transporters [54]. Subsequently, translational control of *AtARFs* by upstream ORF (uORFs) has been proposed as a regulatory mechanism required in modulating auxin responses during plant development [55]. Though direct experimental evidence is still lacking, tomato ARFs may also undergo the same mode of regulation.

In addition of being auxin-responsive, the expression of some *Sl-ARFs* was found to be regulated by ethylene. The presence of auxin and ethylene *cis*-regulatory elements in the promoter region of a number of *Sl-ARFs*, supports the potential regulation of *ARF* genes by both auxin and ethylene and suggests that these transcription factors have the ability to mediate both auxin and ethylene responses. In support to this hypothesis, Arabidopsis *ARF19* has been shown to be inducible by ethylene and has been reported to contribute to ethylene sensitivity through a cross-talk between auxin and ethylene signalling [27], [30]. Also, *ARF2* has been shown to regulate the hook curvature of etiolated Arabidopsis seedlings, a typical ethylene response [27]. Taking together, these data suggest that ARFs may act at the crossroads of auxin and ethylene signaling.

Altogether, the data provide molecular clues on how ARFs can contribute to the specificity and selectivity of auxin responses through (i) structural features, (ii) differential expression of family members at the tissue and organ levels and, (iii) ability to negatively or positively impact transcriptional activity of target genes. The auxin and ethylene regulation of some ARF members suggest their specific role in the multi-hormonal cross-talks. The regulation of the expression of *ARFs* by alternative splicing during fruit set provides new insight into the complexity of regulation of these genes at the post-transcriptional level.

## Materials and Methods

### Plant material and growth conditions

Tomato seeds (*Solanumlycopersicum* cv MicroTom or Ailsa Craig) were sterilized, rinsed in sterile water and sown in recipient Magenta vessels containing 50 mL of 50% Murashige and Skoog (MS) culture medium added with R3 vitamin (0.5 mg L^−1^ thiamine, 0.25 mg L^−1^ nicotinic acid and 0.5 mg L^−1^pyridoxine), 1.5% (w/v) sucrose and 0.8% (w/v) agar, pH 5.9. Plants were grown under standard greenhouse conditions. The culture chamber rooms are set as follows: 14-h-day/10-h-night cycle, 25/20°C day/night temperature, 80% hygrometry, 250 µmol m^−2^s^−1^ intense luminosity.

### *In silico* Identification of the tomato ARFs

All the ARF gene sequences (ITAG2.3_gene_models.gff3) are download from the Sol Genomics Network (http://solgenomics.net/), and analyzed in Notepad++ software. The NLS location was searched using cNLS Mapper (http://nls-mapper.iab.keio.ac.jp/cgi-bin/NLS_Mapper_form.cgi). All the obtained sequences were sorted for the unique sequences and these were further used for B3, AUX_RESP, and Aux/IAA domain search using InterProScan Sequence Search (http://www.ebi.ac.uk/Tools/pfa/iprscan/). The UTR of Sl-ARFs were found by two steps, first, the whole tomato genome and Sl-ARF gene structures (ITAG2.3_gene_models.gff3) were loaded into the Java, and then, the complete cDNA sequences from RNA-Seq data including three stages (flower bud, anthesis and post-anthesis) were blast with Sl-ARF gene structures to identify the final 5′ or 3′ UTRs in Sl-ARFs. The miRNA location on the Sl-ARFs were searched depend on the GBF data (http://tata.toulouse.inra.fr/gbf/blast/blast.html) and SGN Blast tools. Taken together, all of the Sl-ARF family structures were drawn by Fancy Gene v1.4 (http://host13.bioinfo3.ifom-ieo-campus.it/fancygene/) with manual correction.

### Transient Expression Using a Single Cell System

Protoplasts were obtained from suspension-cultured tobacco (*Nicotianatabacum*) BY-2 cells and transfected by a modified polyethylene glycol method as described by Abel and Theologis [56]. For nuclear localization of the selected ARF fusion proteins, the coding sequence of genes were cloned as a C-terminal fusion in frame with GFP under the control of the 35S CaMV, a cauliflower mosaic virus promoter. Transfected protoplasts were incubated for 16 h at 25°C and analysed for GFP fluorescence by confocal microscopy. For co-transfection assays, aliquots of protoplasts (0.5×10^6^) were transformed either with 10 µg of the reporter vector alone containing the promoter fused to the GFP reporter gene or in combination with 10 µg of ARF contructs as the effector plasmid. Transformation assays were performed in three independent replicates. After 16 h, GFP expression was analyzed and quantified by flow cytometry (FACS Calibur II instrument, BD Biosciences, San Jose, CA) on the flow cytometry platform, IRF31, Inserm, Toulouse and and cell sorting platform, INSERM UPS UMR 1048, Toulouse RIO imaging platform. Data were analyzed using Cell Quest software. For each sample, 100 to 1000 protoplasts were gated on forward light scatter and the GFP fluorescence per population of cells corresponds to the average fluorescence intensity of the population of cells after subtraction of autofluorescence determined with non transformed BY-2 protoplasts. The data are normalised using an experiment, in presence of 50 µM 2.4 D, with protoplasts transformed with the reporter vector in combination with the vector used as the effector plasmid but lacking Sl-ARF coding region.

### RNA isolation and Quantitative RT-PCR

Total RNA from fruit was extracted according to the method of Hamilton *et al.* [57]. Total RNA from leaves and seedlings was extracted using a Plant RNeasy Mini kit (Qiagen) according to the manufacturer's instruction. Total RNA was treated by DNase I to remove any genomic DNA contamination. First strand cDNA was reverse transcribed from 2 µg of total RNA using Omniscript kit (Qiagen) according to the manufacturer's instruction.The qRT-PCR analysis was performed as previously described [3]. The sequences of primers are listed in Table S3 in File S1. Relative fold differences were calculated based on the comparative Ct method using the Sl-Actin as an internal standard. To determine relative fold differences for each sample in each experiment, the Ct value of genes was normalized to the Ct value for Sl-Actin-51 (accession number Q96483/Solyc11g005330) and was calculated relative to a calibrator using the formula 2^−ΔΔCt^. At least two to three independent RNA isolations were used for cDNA synthesis and each cDNA sample was subjected to real-time PCR analysis in triplicate. Heat map representation was performed using centring and normalized ΔCt value, with Cluster 3.0 software and Java Tree view to visualize dendogram.

### Hormone treatment

For auxin treatment on light grown seedlings, 12-day-old tomato seedlings (30 seedlings) were soaked in liquid MS medium with or without (mock treatment) 10 µM IAA for 2 hours. The efficiency of the treatment was checked by measuring the induction of the tomato early auxin-responsive SAUR gene. For ethylene treatment on dark grown seedlings, 5-days-old MicroTom seedlings (100 seedlings) were treated with air or ethylene gas (50 µL/L) for 5 hours. The efficiency of the treatment was checked by measuring the induction of the tomato ethylene-responsive E4 gene. Experiment was repeated for 3 biological times.

### RNA-Sequencing and RT-PCR

Total RNA was extracted from bud, flower and post-flower (4DPA) for three biological repeats using a TRIZOL Reagent (invitrogen) according to the manufacturer's instruction. Total RNA was treated by DNase I to remove any genomic DNA contamination and checked by RNA gel and Agilent RNA 6000 Nano Assay, which the RIN value above 7 was determined to be qualified. After that, the best RNA were sent out for deep RNA sequencing using Illumina Hiseq2000 and the reads generated were mapped to the tomato genome sequence SL2.40. The data are desposited at NCBI SRA database under the accession number SRP029978 The gene expression was calculated for each annotated tomato gene (iTAG2.30). For continuous validation, first strand cDNA was synthesized as previously described and PCR was performed using primers designed from the intron and exon of 7 *Sl-ARF* genes. The primer sequences are listed in Table S4 in File S1. An aliquot of 1 ul of the product was used as a template. The PCR amplification cycle was as follows: 95°C for 30 s, 56–60°C for 40 s, 72°C for 30 s-2.5 min. Samples were taken after 25, 30 or 35 cycles and 10 ul of the PCR product was visualized on a 2–2.5% agarose gel. All PCRs were carried out in a Mastercycler (Eppendorf, Hamburg, Germany). DNA was stained with ethidium bromide in the gel. Sl-Ubi3 expression was used as an internal control.

## Supporting Information

File S1 **Supporting tables and figures. Table S1.** uORF prediction in the 5′UTR leader sequences of Sl-ARFs. **Table S2**. *In silico* analysis of *Sl-ARF* gene promoters. **Table S3.** Quantitative RT-PCR primers of *Sl-ARF* genes. **Table S4**. PCR primers for identifying the alternative splicing expressed forms in *Sl-ARF* genes. **Figure S1.** ***Sl-ARF*** **genes genomic distribution on the tomato chromosomes.** The arrows next to gene names show the direction of transcription. The number near to each *Sl-ARF* designates the position megabases (Mb) of the first ATG in the tomato chromosome pseudomolecules (tomato genome version SL2.40). The chromosome numbers and their corresponding size are indicated at the top and bottom of each bar. **Figure S2**. **Phylogenetic relationship between tomato Sl-ARF genes.** The unrooted tree was generated using MEGA4 program by neighbor-joining method. Bootstrap values (above 50%) from 1000 replicates are indicated at each branch. Sl-ARFs with a star (*) are deprived of domain III and IV necessary for interaction with Aux/IAAs. **Figure S3.1-6. Predicted alternative splicing in six Sl-ARFs (Figure S3.1 to Figure S3.6).** RNA-seq reads generated during the fruit-set and mapped on the corresponding *Sl-ARF* gene sequence (*Sl-ARF2B, 3, 4, 8A, 8B*, and *24*) showing predicted alternative splicing events. RNA-seq reads are represented by red and blue rod arrows.
(PDF)
