# Supplementary material for: Characterization of the Tomato ARF Gene Family Uncovers a Multi-Levels Post-Transcriptional Regulation Including Alternative Splicing
Source: PLoS One. 2014 Jan 10;9(1):e84203. doi: 10.1371/journal.pone.0084203 (PMC3888382; doi:10.1371/journal.pone.0084203)

Table S1: uORF prediction in the 5'UTR leader sequences of Sl-ARFs

[a]Gene name,

[b]iTAG release 2.30 name,

[c]predicted uORF number,

[d]size of the corresponding uORFs

[e]sequence of the uORFs

Gene name[a]	ITAG number[b]	uORF number[c]	size uORF (AA) [d]	uORF sequence[e]
Sl-ARF1	Solyc01g103050.2	2	11	mnviyvdydei
			11	mmryseirvnt
Sl-ARF2A	Solyc03g118290.2	5	12	mkkllllsiedr
			13	mnthihtvfrern
			4	mlls
			2	mn
			5	mrdlc
Sl-ARF2B	Solyc12g042070.2	3	52	mvkslfssfsvfspynhspnslkiyrdkvaisadsefvlqfacldclskgfa
			6	mfglfk
			5	mrdlr
Sl-ARF3	Solyc02g077560.2	3	1	m
			1	m
			2	mr
Sl-ARF4	Solyc11g069190.2	4	29	mssassvpssfnskmgvemesyfeflvii
			16	mtflffsveflflfll
			4	milt
			3	mln
Sl-ARF5	Solyc04g081240.2	6	12	mlscsvvcfhss
			16	mfcsmfsfllriiplt
			10	mwvgsyvsln

			3	mlv
			4	mssc
			11	mgslvlvlglm
Sl-ARF6A	Solyc12g006340	3	1	m
			16	mkeltlvvfscgsgvg
			9	mfacflvfv
Sl-ARF6B	Solyc07g043620.3	2	44	msknmvscflvfawgggvgvggctskvliflefllsgvalcwln
			16	mkqllmvvfscgtrfg
Sl-ARF7A	Solyc07g016180.2	2	8	mccvlcvw
			4	mnlr
Sl-ARF7B	Solyc05g047460.3	2	1	m
			4	mnlr
Sl-ARF8A	Solyc03g031970.3	2	9	mevlekekc
			4	mlgs
Sl-ARF8B	Solyc02g037530.3	4	34	mekrwgilkigfqlifgvgiegflqfsgllqrrv
			27	mwgdhihngekvgyledrvsvdfwswn
			7	mevleke
			8	mhvgrsys
Sl-ARF9A	Solyc08g082630.3	1	3	mff
Sl-ARF9B	Solyc08g008380.3	3	23	mqdlefliIklflificdkdtnf
			4	mipi
			4	mkll
Sl-ARF10A	Solyc11g069500.2	7	18	mlltsltcndpsfrccfv
			4	mhlc
			6	mlniyr
			9	mcflklksl
			24	misfffqflrfnisfhnrkfsvsf
			10	mcftisfsvl
			7	mdllccl
Sl-ARF10B	Solyc06g075150.3	1	1	m
Sl-ARF16A	Solyc09g007810.3	5	2	mf
			12	mrsfglnlvklf
			3	myl

			3	mgk
			2	mr
Sl-ARF16B	Solyc10g086130.1	not expressed		
Sl-ARF17	Solyc11g013470.2	not expressed		
Sl-ARF18	Solyc01g096070.3	1	36	mlkcvntengfffklccyrpffvcfgflvkfwvisf
Sl-ARF19	Solyc07g042260.2	0		
Sl-ARF24	Solyc05g056040.2	0		

Table S2: Analysis of *Sl-ARF* gene promoters. The 2.5 Kb promoter fragment of the *Sl-ARF* gene was analyzed *in silico* using PlantCare software (http://www.dna.affrc.go.jp/PLACE/signalscan.html) to search for the presence of the canonical Auxin-Response Element (AuxRE) motif known as TGTCTC-box and the Ethylene-Response Element (ERE) known as AWTTCAA-box. Positions are expressed as negative values with regard to the Star Codon given +1 value.

Gene	*Code*	*Number of the AuxRE (TGTCTC)*	*Number of the ERE*
Sl-ARF1	Solyc01g103050	1	4
Sl-ARF2A	Solyc03g118290	3	4
Sl-ARF2B	Solyc12g042070	1	3
Sl-ARF3	Solyc02g077560	4	1
Sl-ARF4	Solyc11g069190	5	6
Sl-ARF5	Solyc04g081240	0	4
Sl-ARF6A	Solyc12g006340(Nter); Solyc00g196060(Cter)	4	1
Sl-ARF6B	Solyc07g043620	1	1
Sl-ARF7A	Solyc07g016180	0	5
Sl-ARF7B	Solyc05g047460	4	2
Sl-ARF8A	Solyc03g031970	2	1
Sl-ARF8B	Solyc02g037530	0	4
Sl-ARF9A	Solyc08g082630	1	1
Sl-ARF9B	Solyc08g008380	2	2
Sl-ARF10A	Solyc11g069500	1	3
Sl-ARF10B	Solyc06g075150	1	0
Sl-ARF16A	Solyc09g007810	3	2
Sl-ARF16B	Solyc10g086130	0	4
Sl-ARF17	Solyc11g013480(Nter); Solyc11g013470(Cter)	2	1
Sl-ARF18	Solyc01g096070	0	0
Sl-ARF19	Solyc07g042260	0	2
Sl-ARF24	Solyc05g056040	3	2

Table S3: Quantitative PCR primers of Sl-ARFs genes used in this work.

Gene name	Name in iTAG release 2.30	Forward primer	Reverse primer
Sl-ARF1	Solyc01g103050	TCTCCTTCATCATTCTCATACTG	GAACCATTCTCACCATAACC
Sl- ARF 2A	Solyc03g118290	GCAAGGTCAAGAGTTATCGA	CATTGGTTTCTCAGACAAGTC
Sl- ARF 2B	Solyc12g042070	CACTTAATCCACTTCCAATACC	TACAACTACTTTGGATGAACCT
Sl- ARF 3	Solyc02g077560	AATTGCAGTATCAGACTTTGG	TCTAGATATCCCAGAACTAGGA
Sl- ARF 4	Solyc11g069190	CATTATTGTTGGTGACTTTGTG	GACCTTTGGAAACCTATTGG
Sl- ARF 5	Solyc04g081240	CCTTCAGAGTTTGTCATTCCT	AACATCATTCCAAATCTCATACC
Sl- ARF 6A	Solyc12g006340(Nter); Solyc00g196060(Cter)	CCAACATATCCCTAGTACTTCAG	GTGCCTGAGATATTAGTTGGT
Sl- ARF 6B	Solyc07g043620	ACCCTCTAGTATCTTCATCCT	TCCGAGACCTTTGTATTGTG
Sl- ARF 7	Solyc07g016180	TCAACTCCTCAAACATACCT	TGAACTATCCAAATAATCCATCTG
Sl- ARF 8A	Solyc05g047460	TGACATCGAATGGAAATTCAG	GTCTCTTAGCACTAACAAACAC
Sl- ARF 8B	Solyc03g031970	GTCAGTCCGTGATCATAGAG	GGAATCCAAGCTACAATTTCC
Sl- ARF 9	Solyc02g037530	ATCATTCAATCTCAAATCAAAGGT	CCTCATCATTGTCTTCTTCAG
Sl- ARF 10	Solyc11g069500	ATTCTCTGTGCCTAGATACTG	CTATAAATGTGCCTAAACTTCCA
Sl- ARF 17	Solyc11g013480(Nter); Solyc11g013470(Cter)	TGAAGTTGATGAAGTTACTATGAG	TCCTCCATTATTCGCATCTG
Sl- ARF 18	Solyc01g096070	AATCTACACTCGGCATTGTC	AAGCTTCCTATCTTATCATTGGA
Sl- ARF 19	Solyc07g042260	TGGTGGATGAATCTGTTGTC	TACTTAGACAGCTCTGAACCT
Sl- ARF 24	Solyc05g056040	TCATTGTTGGATGTTTCAAAGG	GAAGTCTTGGAAAGTAGTATACTC
Sl-Actin	Solyc11g005330	TGTCCCTATCTACGAGGGTTATGC	AGTTAAATCACGACCAGCAAGAT

Table S4: PCR primers for identifying the alternative splicing expressed forms in *Sl-ARF* genes.

		Primers sequence 5'-3'	
**Gene name**	**Details**	**Forward**	**Reverse**
Sl-ARF2B	Intron 11	AACCTTAAGAAAGTGCCAAAAGTAC	ATAACTTGCCCTTATATTTGAATTCC
Sl-ARF3	Intron 9	AATTCTGAAATACTCCCTCCGT	AGCATATCCAGACCTAGTCTCCA
Sl-ARF4	Intron 6	GCACAGAGTGAAAGATTTGGG	GCCAAACCATCAATTATCTTCC
Sl-ARF8A	Intron 6	AAGTTGTTATACAGTGGGTCAAGG	GCAAATCTCACTCACAATGTCAG
Sl-ARF8B	Intron 11	CGCTGAATGGTCATGTAATAAGAG	ATTCCTTCTATCATGGCTATCTGG
Sl-ARF19	Intron 1	ATAAGGAGTTCTGCAAGCCA	GTAGGTTGTGCTATGCTGAC
Sl-ARF24	Intron 3	GGACAATATGTAGCAATTAGGGAC	AGACCATGTGATTTGAGTACCA
Sl-ARF2B	Ex 11-Int 11-Ex 12	AACATCAGCCTTCTCGTCATCC	GCACTTAATCCACTTCCAATACCA
Sl-ARF3	Ex 8-Int 9-Ex 9	TAGATCCAGTTCGATGGCCAG	CTCAATCTCCCATGGTGAAACC
Sl-ARF4	Ex 6-Int 6-Ex 7	CTTGTCCCAACAGGAATCCGA	GGAATAAGAAGAGCTGCAAGACCT
Sl-ARF8A	Ex 6-Int 6-Ex 7	TGACATCGAATGGAAATTCAGG	CGGCAACAAGTCTCTTAGCA
Sl-ARF8B	Ex 11-Int 11-Ex 12	GCCTTTCTATCAAGGAACCTC	GAGAATTGTTGAATCGACTCTC
Sl-ARF19	Ex 3-Int 3-Ex 4	GCTCACTGTGACCTTGAGGA	AGGTGAAAGTAGCTTTGTGTTGG
Sl-ARF24	Ex 1-Int 1-Ex 2	GGTCTCAGCATAAACCTCTTCC	GGAGCAATCATCCAACCAGGA
Sl-Ubiquitin		CTAACGGGGAAGACGATCACCC	TCCCAAGGGTTGTCACATACATC

Figure S1

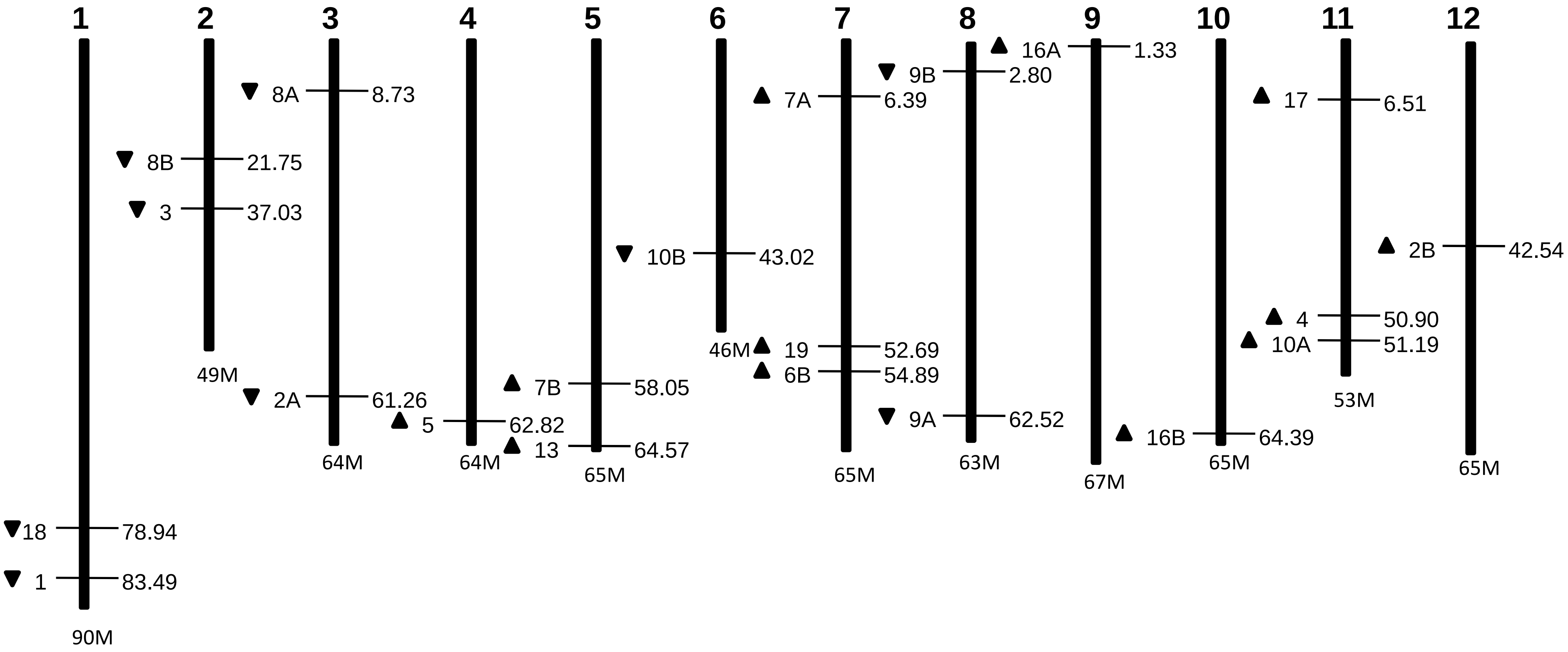
1
2
3
4
5
6
7
8
9
10
11
12
8A
8.73
8B
21.75
3
37.03
49M
2A
61.26
64M
5
62.82
64M
7B
58.05
13
64.57
65M
10B
43.02
46M
7A
6.39
19
52.69
6B
54.89
65M
9B
2.80
9A
62.52
63M
16A
1.33
67M
16B
64.39
65M
17
6.51
4
50.90
10A
51.19
53M
2B
42.54
65M
18
78.94
1
83.49
90M

Figure S2

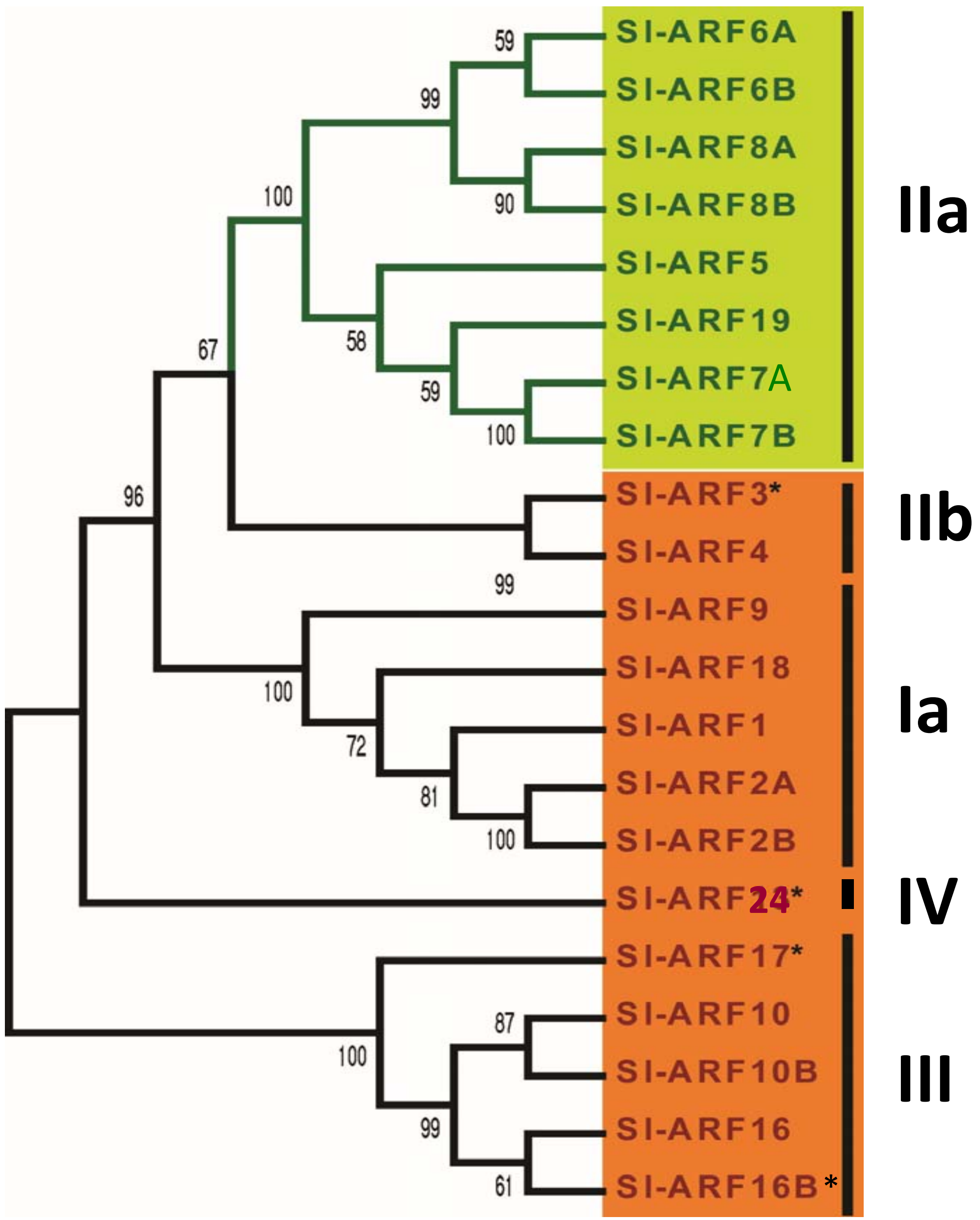

SI-ARF6A
SI-ARF6B
SI-ARF8A
SI-ARF8B
SI-ARF5
SI-ARF19
SI-ARF7A
SI-ARF7B
SI-ARF3*
SI-ARF4
SI-ARF9
SI-ARF18
SI-ARF1
SI-ARF2A
SI-ARF2B
SI-ARF24*
SI-ARF17*
SI-ARF10
SI-ARF10B
SI-ARF16
SI-ARF16B*
IIa
IIb
Ia
IV
III
59
99
90
100
58
59
100
67
96
99
100
72
81
100
87
100
99
61

Figure S3.1

Sl-ARF2B

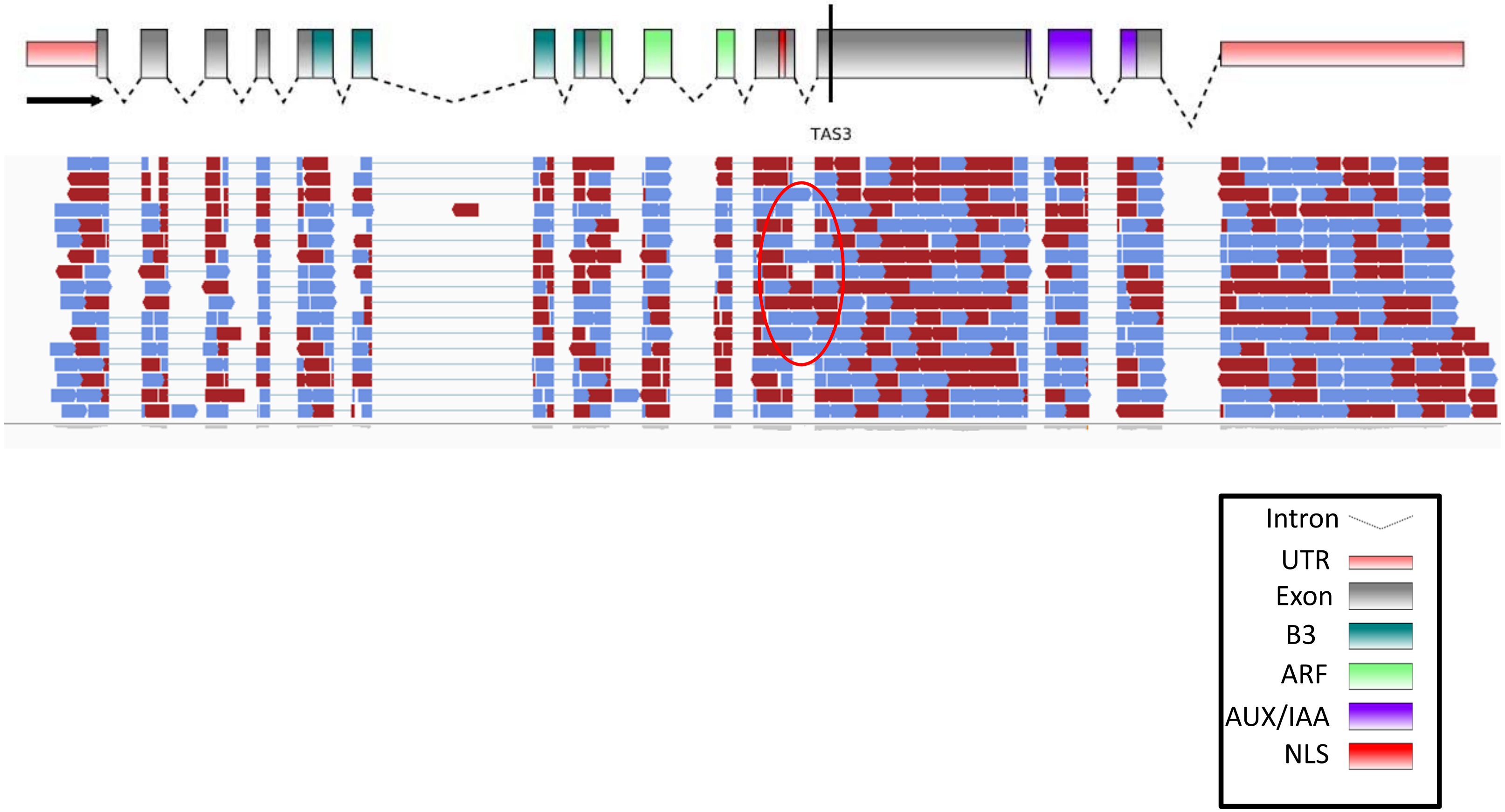

Figure S3.2

Sl-ARF3

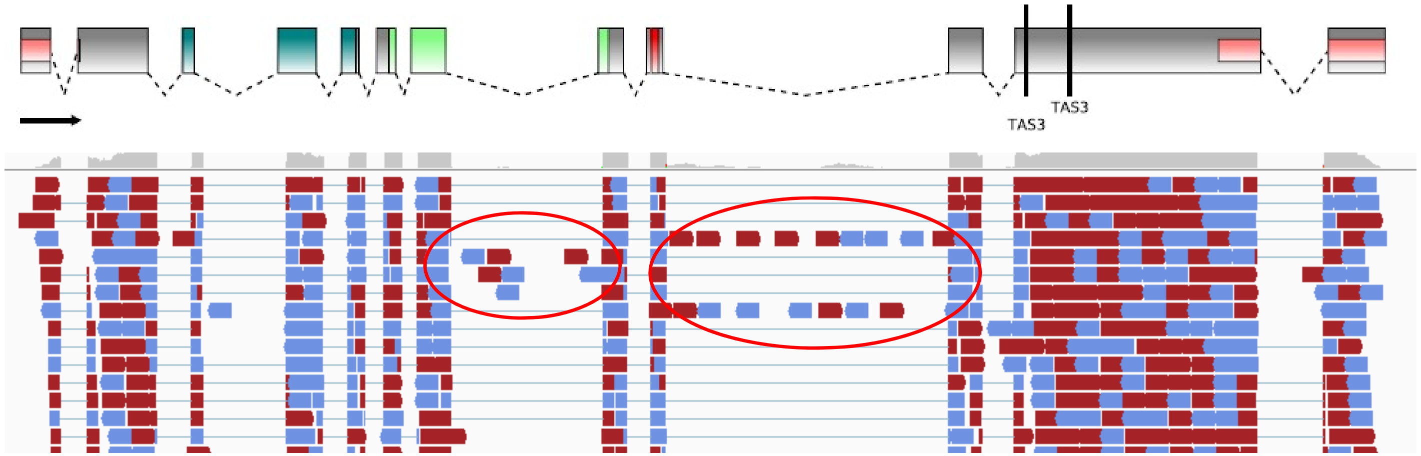

Figure S3.3

Sl-ARF4

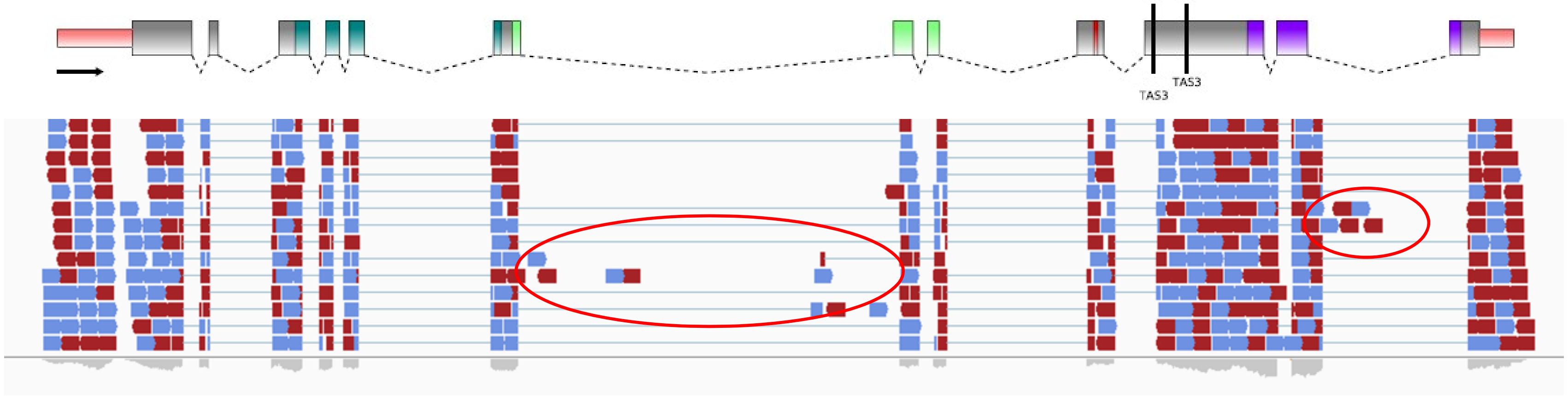

Figure S3.4

Sl-ARF8A

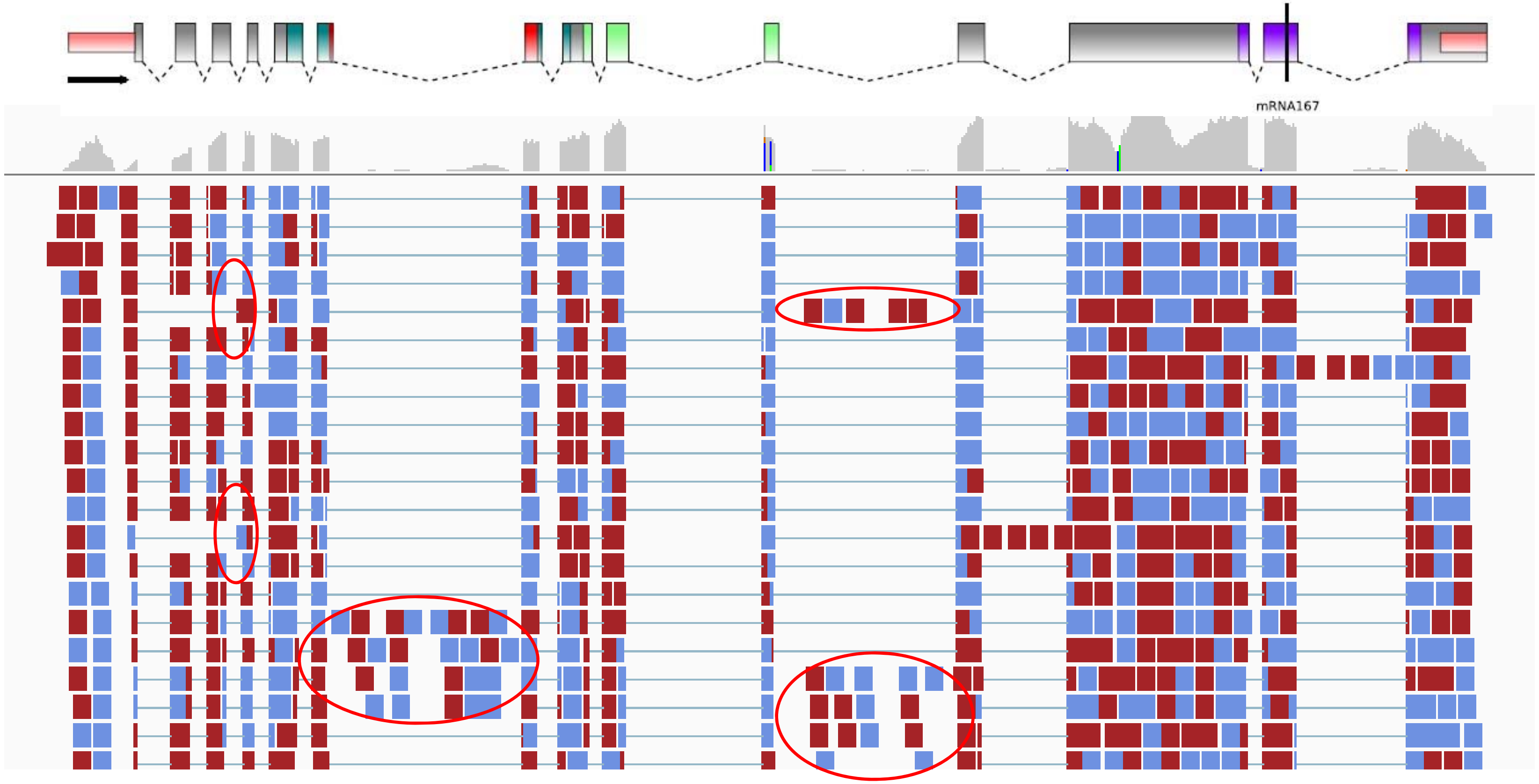

Figure S3.5

Sl-ARF8B

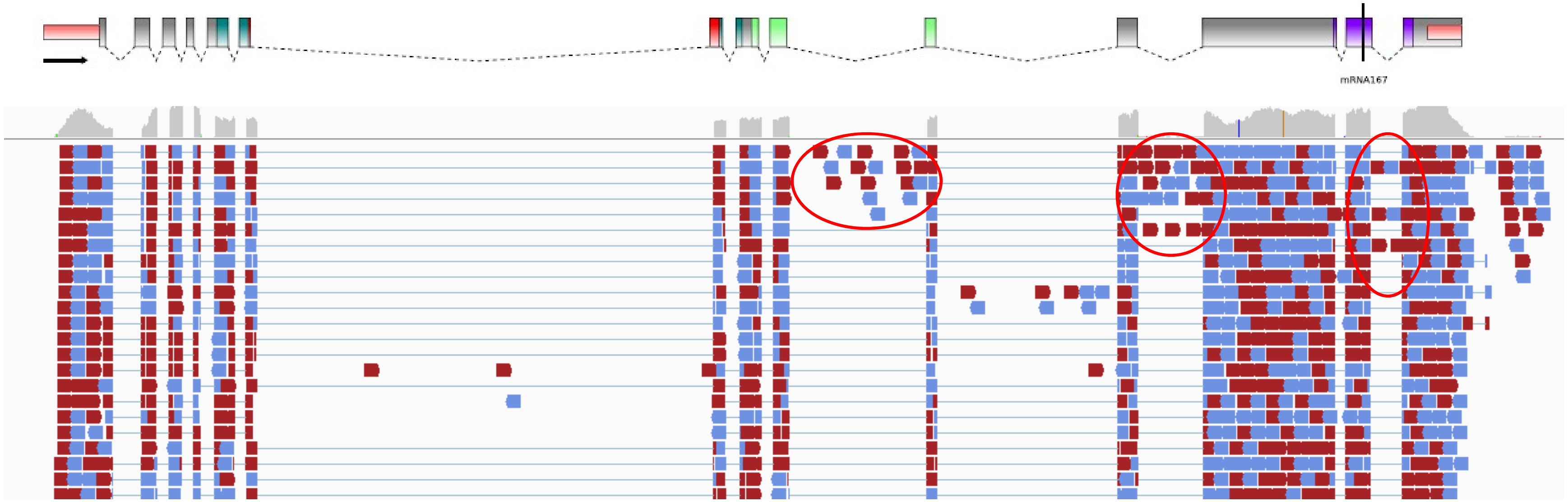

Figure S3.6

Sl-ARF24

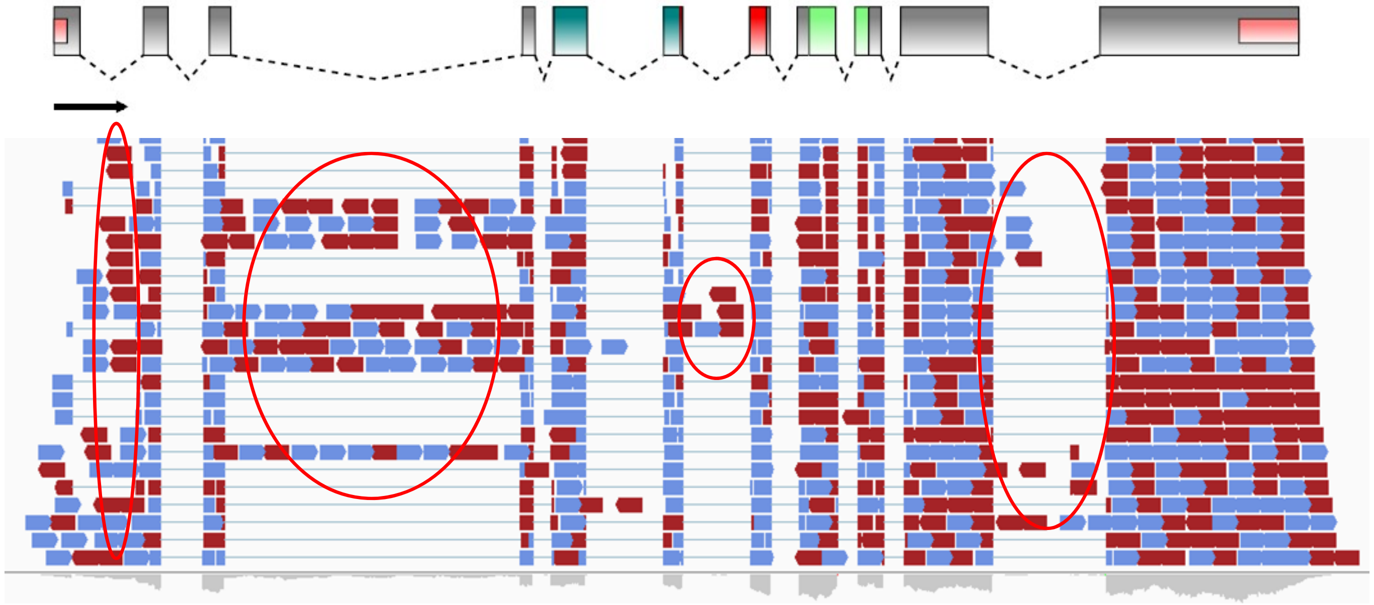

Supplement: File S1 — Supporting tables and figures. Table S1. uORF prediction in the 5′UTR leader sequences of Sl-ARFs. Table S2. In silico analysis of Sl-ARF gene promoters. Table S3. Quantitative RT-PCR primers of Sl-ARF genes. Table S4. PCR primers for identifying the alternative splicing expressed forms in Sl-ARF genes. Figure S1. Sl-ARF genes genomic distribution on the tomato chromosomes. The arrows next to gene names show the direction of transcription. The number near to each Sl-ARF designates the position megabases (Mb) of the first ATG in the tomato chromosome pseudomolecules (tomato genome version SL2.40). The chromosome numbers and their corresponding size are indicated at the top and bottom of each bar. Figure S2. Phylogenetic relationship between tomato Sl-ARF genes. The unrooted tree was generated using MEGA4 program by neighbor-joining method. Bootstrap values (above 50%) from 1000 replicates are indicated at each branch. Sl-ARFs with a star (*) are deprived of domain III and IV necessary for interaction with Aux/IAAs. Figure S3.1-6. Predicted alternative splicing in six Sl-ARFs (Figure S3.1 to Figure S3.6). RNA-seq reads generated during the fruit-set and mapped on the corresponding Sl-ARF gene sequence (Sl-ARF2B, 3, 4, 8A, 8B, and 24) showing predicted alternative splicing events. RNA-seq reads are represented by red and blue rod arrows. (PDF) [file pone.0084203.s001.pdf]
